# Outcomes of total arterial revascularization vs conventional revascularization in patients undergoing coronary artery bypass graft surgery: A narrative review of major studies

**DOI:** 10.12669/pjms.38.5.5674

**Published:** 2022

**Authors:** Carmelo Dominici, Massimo Chello, Sahrai Saeed

**Affiliations:** 1 Carmelo Dominici, MD, Department of Cardiac Surgery, Università Campus Bio-Medico di Roma, Rome, Italy; 2 Massimo Chello, MD, Department of Cardiac Surgery, Università Campus Bio-Medico di Roma, Rome, Italy; 3 Sahrai Saeed, MD, PhD, FESC. Department of Heart Disease, Haukeland University Hospital, Bergen, Norway

**Keywords:** Total arterial revascularization, Internal mammary artery, Radial artery, CABG

## Abstract

Coronary artery bypass grafting (CABG) is a widely used surgical procedure which improves clinical outcomes in appropriately selected patients. Conventionally, the greater saphenous vein is often used in CABG. However, due to their higher long-term patency rates, arterial conduits are routinely used, with the left internal thoracic artery (LITA) on left anterior descending (LAD) being the gold standard in CABG. Our aim in the present work was to investigate the outcomes of a total arterial grafting (TAG) on the whole heart, with no use of venous grafts, compared to mixed conduits in real-world data. A literature search was conducted in the bibliographic databases PubMed and Web of Science. Only studies comparing TAG with conventional CABG (at least one venous graft plus one or more arterial grafts), with at least one hundred patients in each group were included in this review. After study selection, a total of 15 relevant studies were evaluated and discussed in the present review. Results indicated that TAG is a highly efficient technique, and multiple arterial grafts can be used to reliably revascularize all coronary artery territories. TAG was more beneficial in terms of both short and long-term outcomes and its use should be encouraged. Large randomized clinical trials are needed to confirm the superiority of total arterial grafting with regard to long-term outcomes.

## INTRODUCTION

Coronary artery bypass grafting (CABG) is the most common cardiac surgery performed worldwide, with around 800000 cases annually around the globe.^1^ It is the optimal revascularization strategy for multi-vessel coronary disease, both in terms of symptom relief and prognosis.^2^ Although the overall complication rates, per-operative parameters such as perfusion time and intensive care unit stay have improved over the years, the non-risk stratified mortality differs between developed nations and South Asian countries with slightly higher rates in the latter.^3^ Avoidance of recurrent angina is the aim of long-term relief, and the choice of conduits plays a critical role in this.^4^ It is well-proven that the patency of arterial grafting is superior to that of saphenous veins, especially in terms of long-term survival.^2^,^5^ The use of LITA (Left Internal Thoracic Artery) on LAD (Left Anterior Descending) system is the gold standard in CABG and shown to improve survival.^2^ However, on the rest of the heart, it is still a debate. On the right system of the heart, the European guidelines recommend the use of arterial graft in young patients or patients with poor veins or advanced coronary disease.^2^,^6^ Despite better patency, arterial grafting has still not become the standard of care. The Arterial Revascularization Trial (ART) has shown the benefit of using BITA vs LITA (two arteries on the left system compared to one). However, the rest of the heart was left to venous grafts or a third conduit.^7^,^8^ This study aims at investigating the outcomes of a total arterial grafting (TAG) on the whole heart, with no use of venous grafts, compared to mixed conduits (LITA/BITA plus at least one venous conduit) in real-world data, without considering results from small retrospective studies with less than 100 patients per study arm.

## METHODS

A literature search was conducted using PubMed and Web of Science in October 2021. 1185 results were obtained in which the key words “total arterial revascularization” and “CABG” were mentioned either in the title or abstract. Long-term cardiac survival was the primary endpoint. Secondary endpoints were short-term survival (30-day mortality/in-hospital mortality), major adverse cardiac or cerebrovascular events (MACCEs) and deep sternal wound infections (DSWI).

Notably, only studies comparing TAG (multiple arterial grafts with no venous grafts) with conventional CABG (at least one venous graft plus one or more arterial grafts), with at least one hundred patients in each group, were included in this review. After study selection, a total of 15 papers were evaluated in detail and are discussed below (Table-I).

**Table I:** Studies included in the review.

Study	Year	PSM overall cohort, patients	TAG, patients	TAG, conduits	Conventional, patients	Conventional, conduits	Outcomes
Rocha et al.^10^	2020	4264	2132	LITA+RITA, LITA+RITA+RA	2132	LITA/BITA+SVG	In-hospital mortality, 8-yr survival and MACCEs
Di Bacco et al.^19^	2020	718	359	LITA+RITA, LITA+RITA+RA	359	LITA+SVG	In-hospital mortality, 10-yr mortality
Formica et al.^18^	2019	380	190	LITA+RITA+RA	190	LITA+RITA+SVG	In-hospital mortality, 15-yr mortality
Royse et al.^17^	2018	464	232	LIMA+RA	232	LITA+SVG	21-yr survival (Kaplan Meier figures only)
Malik et al.^20^	2017	380	190	LITA+RITA	190	LITA+SVG	30-days mortality
Mohammadi et al.^27^	2016	498	249	LITA+RITA+RA	249	LITA+RITA+SVG	In-hospital mortality, 15-yr mortality
Bisleri et al.^11^	2016	302	151	LITA+RITA, LITA+RITA+RA	151	LITA+SVG	In-hospital mortality
Shi et al.^16^	2015	524	262	LITA+RITA+RA	262	BITA+SVG	30-days mortality, 15-yr survival
Tatoulis et al.^9^	2015	12464	6232	LITA+RITA+RA	6232	LITA/RITA/RA+SVG	30-days mortality, long-term survival (ACM)
Garatti et al.^12^	2013	452	209	Mixed	243	LITA+SVG	In-hospital mortality, long-term survival
Grau et al.^13^	2012	1856	928	LITA+RITA	928	LITA+SVG	30-days morbidity and mortality, long-term survival
Buxton et al.^14^	2012	206	103	LITA+RITA, LITA+RITA+RA	103	LITA/RITA/RA+SVG	30-days survival, 12-years survival
Hassanein et al.^21^	2010	804	289	LITA+RITA	415	LITA+SVG	30-days morbidity and mortality
Muneretto et al.^15^	2006	200	100	LITA+RITA+RA	100	LITA+SVG	In-hospital mortality, 34 months follow up
Baskett et al.^28^	2006	4452	2226	LITA+RITA+RA, RITA+RA (AA)	2226	LITA/RITA/RA+SVG (A1V)	In-hospital mortality

Only studies comparing TAG (multiple arterial grafts with no venous grafts) with conventional CABG (at least one venous graft plus one or more arterial grafts), with at least 100 patients in each group, were included in this review.

PSM= propensity score matched; TAG= Total Arterial Grafting; LITA=Left Internal Thoracic Artery; RITA= Right Internal Thoracic Artery;

BITA= Bilateral Internal Thoracic Artery; SVG= Saphenous vein graft; RA= Radial artery; ACM = All-cause Mortality; AA=All arterial; A1V=One arterial + veins.

## RESULTS

All studies reported short-term mortality which was defined as either in-hospital mortality or 30-day mortality. There was a trend in superiority of TAG compared to SVG for short-term survival and Tatoulis et al. reached statistical significance in favor of TAG (0.9% vs 1.2%, P<0.001).^9^ Recently, Rocha et al.^10^ reported a propensity score matched cohort of more than 4000 patients, concluding that in-hospital mortality was similar between TAG and conventional CABG. Over a follow up of eight years, TAG improved freedom from MACCEs (hazard ratio [HR] 0.78, 95% CI 0.68-0.89) and overall survival (HR for death 0.80, 95% CI 0.66-0.97).^10^

Long-term cardiac survival was reported by most studies.^9^,^11^-^18^ Survival estimate at different time intervals during follow-up in patients undergoing TAG or conventional CABG, reporting survival from cardiac-related mortality is shown in Table-II. All reported a general trend of benefit with TAG especially at 10 and 15 year follow-up, with most studies reaching statistical significance.^9^-^11^,^13^,^15^-^17^,^19^

**Table II:** Reported median long-term survival in the qualitative analysis.

Study	Group	5 years	10 years	15 years
Rocha et al.^10^	TAG	93%	86%	
	Conventional	91%	83%	
Di Bacco et al.^19^	TAG	97%	92%	
	Conventional	94%	87%	
Formica et al.^18^	TAG	95%	84%	79%
	Conventional	96%	85%	80%
Royse et al.^17^	TAG	87%	70%	50%
	Conventional	86%	62%	40%
Mohammadi et al.^27^	TAG	98%	92%	92%
	Conventional	96%	93%	87%
Bisleri et al.^11^	TAG	96%	77%	
	Conventional	84%	72%	
Shi et al.^16^	TAG		90%	82%
	Conventional		81%	72%
Tatoulis et al.^9^	TAG	91%	85%	
	Conventional	90%	81%	
Garatti et al.^12^	TAG	97%		82%
	Conventional	93%		79%
Grau et al.^13^	TAG	96%	89%	79%
	Conventional	91%	79%	61%
Buxton et al.^14^	TAG		69%	
	Conventional		59%	

Most studies described MACCEs either as a group of events^11^ or as individual cardiac/cerebrovascular events (myocardial infarction, atrial fibrillation, arrhythmias, stroke, neurological complications, angina recurrence, repeat PCI or need for coronary reintervention).^9^,^10^,^12^-^15^,^20^,^21^ Most showed no statistically significant difference between the two groups. However, Malik et al. showed a significantly lower occurrence of neurological complications in the TAG arm (0.5% vs 3.7%, P=0.017).^20^ This evidence was supported by Hassanein et al, in which a similar trend was observed (0.0% in TAG vs 1.5% in SVG, P=0.0111).^21^ Muneretto et al. reported significantly lower incidence of cardiac events in the TAG arm (recurrent MI, 7% vs 18%, P=0.03; late MI, 2% vs 10%, P=0.03; need for coronary intervention, 1% vs 12%, P=0.004).^15^

Overall, total arterial technique was shown to reduce the occurrence of MACCEs. Seven studies reported deep sternal wound infection (DSWI) as a separate value^9^,^11^,^13^-^15^,^20^,^21^ while in Garatti et al, DSWI was included in post-operative complications.^12^ All showed no statistical difference between the two groups. The number of diabetic patients in each study is reported in Table-III to annex to this comparison.

**Table III:** Number of diabetic patients in each cohort.

Study	Diabetic patients, TAG group	Diabetic patients, conventional group
Rocha et al.^10^	627 (29%)	639 (30%)
Di Bacco et al.^19^	136 (38%)	133 (37%)
Formica et al.^18^	41 (21%)	34 (18%)
Royse et al.^17^	40 (17%)	38 (16%)
Malik et al.^20^	107 (56%)	104 (56%)
Mohammadi et al.^27^	34 (14%)	30 (12%)
Bisleri et al.^11^	38 (25%)	41 (27%)
Shi et al.^16^	34 (13%)	34 (13%)
Tatoulis et al.^9^	2017 (32.4%)	1967 (31.6%)
Garatti et al.^12^	31 (15%)	34 (14%)
Grau et al.^13^	100 (10.8%)	101 (10.9%)
Buxton et al.^14^	103 (100%)	103 (100%)
Hassanein et al.^21^	131 (31.5%)	111 (28.5%)
Muneretto et al.^15^	100 (100%)	100 (100%)
Baskett et al.^28^	329 (14.8%)	363 (16.3%)

### TAG outcomes in subgroups:

The studies of Buxton et al. and Muneretto et al. included exclusively patients with diabetes, reporting the outcomes on 403 patients.^14^,^15^ Analysis of short-term outcomes revealed no statistical difference between the two groups in both studies. However, long-term survival was significantly better in the TAG arm (90 ± 3.7% vs 81 ± 4.9%, P=0.021 at 10-years; 82 ± 5.2% vs 72 ± 6.0%, P=0.021 at 15-years).^16^ At 34 months, Muneretto et al. reported better patency in the TAG arm (96.4% vs 83.2%, P=0.02) and a lower incidence of cardiac related events as reported earlier.^15^ In diabetic patients, subanalysis of the study of Di Bacco et al.^19^ concluded that long-term survival following TAG is remarkably improved compared to conventional CABG especially in diabetic patients; among diabetics, vein grafts was the strongest predictor of MACCEs (HR 2.41; 95% CI 1.27–4.59, P=0.007) and cardiac mortality (HR 3.24; 95% CI 1.69–6.23, P=0.001).^19^

Hassanein et al. compared patients above 65 years between the two cohorts with a mean age of 72 years. No significant difference was found between the two groups in short-term outcomes. Furthermore, the incidence of post-operative stroke was lower with BITA (TAG), (0% vs 1.5%, P=0.0111).^21^

## DISCUSSION

The search for the best graft selection produced many observational studies. A biological hypothesis supporting TAG is the incremental harm associated with SVG considering their modes of failure, with accelerated atherosclerosis and flow differences with arterial grafts.^10^,^22^ A hypothesized advantage of CABG is the protection against flow-limiting lesions, occlusion, or acute thrombosis of non–flow-limiting lesions.^23^ The clinical benefits related to the use of arterial grafts exclusively in CABG is well-supported by retrospective evidence. The long-term survival advantage of total arterial grafting has been ubiquitous in this review. The benefits of this technique are not different in diabetic patients and thus, it is a safe approach in this group of patients. Surgeons tend to apply this technique most commonly in younger patients,^21^ as also supported by current guidelines.^2^ However, Hassanein et al. has demonstrated that total arterial revascularization can be safely performed in patients older than 65 years. Indeed, the T-graft configuration is beneficial in this age group since it avoids aortic manipulation, which is an important risk factor for post-operative stroke.^21^ The use of total arterial grafting was not statistically associated with DSWI in this study. However, this might be related to the fact that multiple arterial grafts were used in the control group as well. Therefore, the risk of exposure might have been similar in both cohorts. This might need further analysis since it is the most feared complication in this approach.

Three recent meta-analysis have been published in this topic.^4^,^5^,^24^ In a recent meta-analysis that pooled 12 small matched/adjusted observational studies of 33597 patients, TAG was associated with reduced all-cause mortality compared with conventional CABG (HR 0.85, 95% CI, 0.81-0.89).^4^ Other authors, including different studies, concluded that TAG with BIMA may offer a higher protective long-term survival effect at the expense of a higher risk of DSWI (relative risk 1.44, 95% CI 1.17-1.77).^5^ The most recent papers summarizing outcomes of 14 studies and 22746 patients (8941 TAG and 13805 non-TAG) showed that the pooled hazard ratio for long-term mortality (>10 years) was lower in TAG (HR 0.68; 95 %CI 0.59-0.78, P<0.001),^24^ but prospectively hi-quality evidences are still awaited to confirm those findings. An ongoing trial with a target sample size of 4300 patients comparing multiple arterial graft with single arterial graft, the Randomization of Single vs Multiple Arterial Grafts (ROMA) trial (NCT03217006) will hopefully provide a definitive answer as to whether using more arterial grafts will lead to better survival and lower number of adverse events in the long term.^25^

## CONCLUSION

Total arterial grafting is a highly efficient technique, and multiple arterial grafts can be used to reliably and flexibly revascularize all coronary artery territories. Total arterial grafting is more beneficial in both short and long-term outcomes and its use should be encouraged.^4^,^5^,^24^,^26^ This technique minimizes the risk of stroke and has better patency. Drawbacks of this approach include DSWI and longer procedural time, as total arterial grafting is a more challenging surgical technique. However, the improved survival outcomes associated with it makes the additional time and effort worthwhile,^8^ although large randomized clinical trials are needed to confirm the superiority of total arterial grafting with regard to all long-term outcomes.

### Authors’ Contribution:

**CD & MC:** Prepared the first draft which was subsequently revised for important scientific content by **SS**. All authors approved the final submission.
